# Metastatic Renal Cell Cancer Instigating Paraplegia in a Male Patient

**DOI:** 10.7759/cureus.26696

**Published:** 2022-07-09

**Authors:** Ahmed A Al Rashed, Qasim M Isa

**Affiliations:** 1 Urology, Salmaniya Medical Complex, Manama, BHR

**Keywords:** lumbar spinal stenosis (lss), pathological lumbar fracture, urology and oncology, acute paraplegia, renal cell carcinoma (rcc)

## Abstract

Renal cell carcinoma (RCC) constitutes about 2% of all adult malignancies and is the most common malignant renal neoplasm with bony metastases occurring in up to 50% of patients with RCC. In this case, we report a 42-year-old male who presented with chronic back pain and had a sudden episode of paraplegia. The patient was initially referred to the orthopedics service. He had a lumbar X-ray done followed by a CT of the spine that showed a burst fracture of the L1 vertebra with incidental finding of a right renal mass suspicious of RCC. Upon further investigations, the patient was found to have a large heterogeneous renal cortical mass with multiple cystic changes and necrosis invading the Gerota’s fascia as well as a tumor thrombus extending into the right renal vein and inferior vena cava.

Although it has been well established that RCC metastasizes to bones and it is not uncommon for vertebral column involvement, sudden paraplegia and incontinence resulting from lumbar fracture due to metastatic RCC has not been widely published. Conclusively, RCC is a common malignancy in which a significant number of patients have metastatic disease upon presentation and this can lead to initial confusion and delay in diagnosis, hence it should be part of the differential diagnosis when investigating chronic bony pain and pathological fractures.

## Introduction

Renal cell carcinoma (RCC) constitutes about 2% of all adult malignancies and is the most common malignant renal neoplasm, reported to account for 85% of all renal malignancies [1]. The usual sites of metastasis in RCC are the lungs, liver, bone and brain with bony metastases occurring in up to 50% of patients with RCC [1,2]. Almost 15% of bony metastases from RCC are in the spine with the incidence of spinal compression reported occurring in 5% to 14% with metastatic spinal compression [3].

In this case, we report a 42-year-old male who presented with chronic back pain and had a sudden episode of paraplegia whilst using the restroom only to have an incidental RCC noted on a spinal CT scan.

## Case presentation

A previously medically free 42-year-old male presented to the emergency department (ED) with a history of lower back pain that has been present for two months. He reported the pain to be continuously present, increasing in severity over time, and partially relieved by non-steroidal anti-inflammatory drugs (NSAIDs). He denied any history of trauma or other injuries preceding the onset of the pain. He also denied any changes in urine and had no constitutional symptoms such as fever or night sweats. However, he reported a recent loss of weight of about 7 Kg. Consequently, he presented to the ED due to an increase in the severity of the pain, and whilst in the ED he used the restroom and reported that once he attempted to get up from the toilet, he felt a sudden increase in pain with an inability to move both lower limbs associated with urine incontinence.

**Figure 1:**
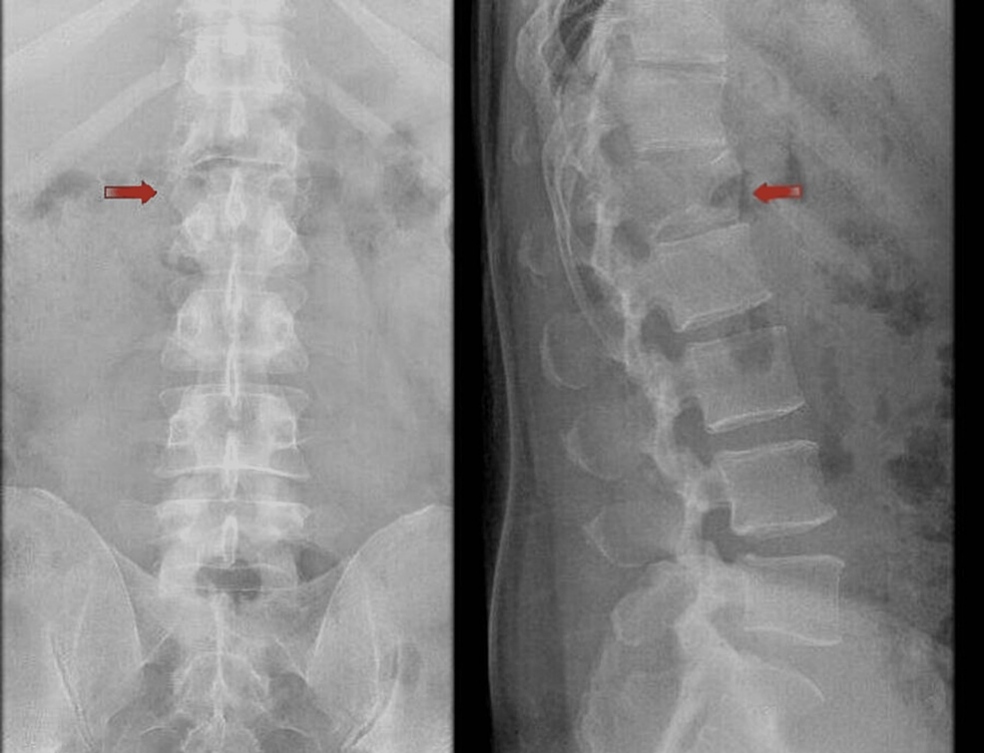
Lateral and anterior-posterior lumbar X-ray demonstrating L1 fracture

The patient was initially referred to the orthopedics service and upon examination, showed a power of 1/5 in all muscle groups from hips below with decreased sensation and decreased overall lower limbs below inguinal ligament as well decreased reflexes and had no anal tone on digital rectal examination. A lumbar X-ray was done in the ED that showed a lumbar fracture at the level of L1 (Figure 1).

**Figure 2:**
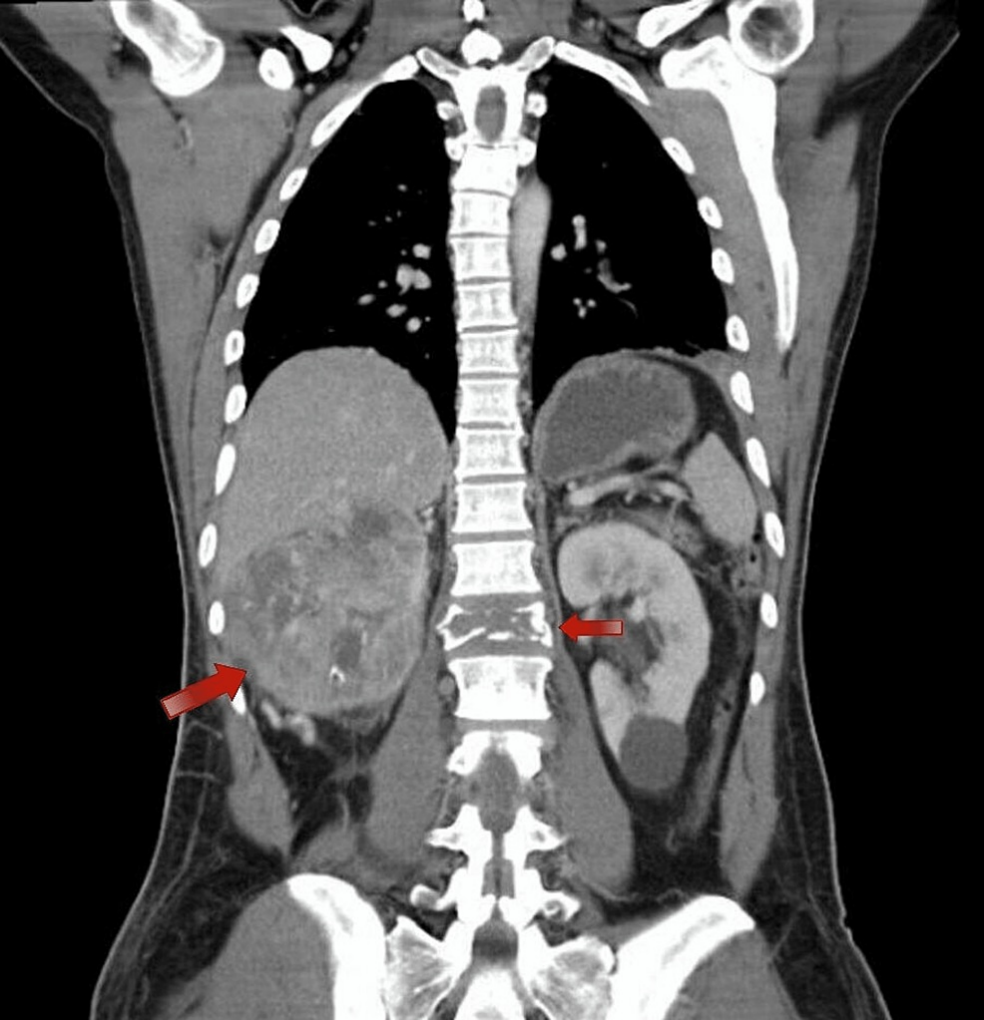
Coronal view of CT abdomen with IV contrast enhancement demonstrating right renal mass with concurrent L1 vertebrae fracture

**Figure 3:**
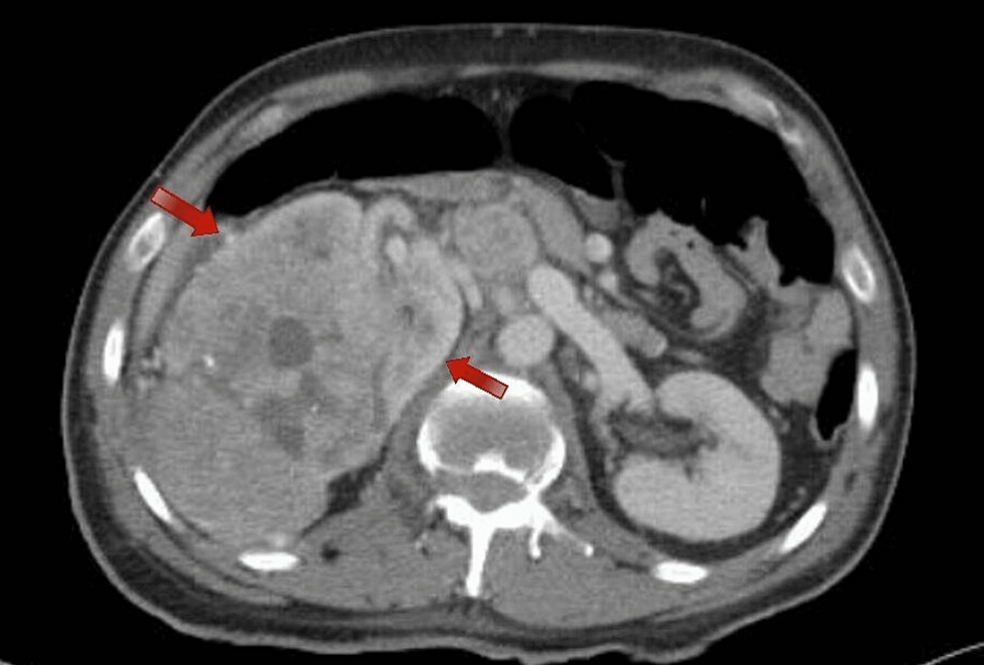
Axial view CT Abdomen with IV contrast enhancement demonstrating right renal mass

Following this finding, the patient was admitted under orthopedics service care and a CT of the spine was done and showed a burst fracture of the L1 vertebra with no gross retropulsed fragment seen in the spinal canal, and part of the posterior kidney was shown and demonstrated an incidental finding of an ill-defined right renal mass with multiple calcifications. Once these findings were noted, a further CT of the abdomen, chest and pelvis with IV contrast was requested and showed a large heterogenous renal cortical mass with multiple cystic changes and necrosis invading the Gerota’s fascia as well as a tumor thrombus extending into the right renal vein and inferior vena cava (Figure 2 and Figure 3). Furthermore, a distal left small pulmonary artery embolism was noted. 

Consequently, the urology team was involved and the patient initially underwent surgical decompression and posterior stabilization with partial extraction of bone fragments of the L1 vertebra with the release of stenosis. Moreover, the patient underwent a needle biopsy procedure for the renal mass and histopathology confirmed the diagnosis of clear cell renal cell carcinoma. Currently, the patient is recovering post-op with physiotherapy for mobilization. However, upon follow-up, he is still unable to mobilize the lower limbs and has a significant motor weakness.

Finally, the case was discussed with the National Tumor Board multidisciplinary team and it was agreed upon that the patient would undergo cytoreductive right nephrectomy and IVC thrombus extraction with anterior stabilization of the L1 vertebra, and then followed up by the oncology team for medical management. 

## Discussion

The most common symptoms that patients with RCC present with are hematuria, flank pain, and a renal mass on palpation. However, since it has been reported that almost a third of patients with RCC have metastatic disease at presentation, various presentations have been reported depending on either the site of metastasis or RCC accompanying paraneoplastic syndromes [4].

Although it has been well established that RCC metastasizes to bones and it is not uncommon for vertebral column involvement, sudden paraplegia and incontinence resulting from lumbar fracture due to metastatic RCC has not been widely published. 

It is known that skeletal metastasis is very destructive in patients with renal cell carcinoma and leads to compromising bone integrity. This can lead to various skeletal-related incidents such as pain, nerve compression, hypercalcemia, and even pathological fractures which may require surgical interventions and other therapy such as in this case [5]. Similarly, cases have been previously reported involving pathological fractures in unlikely locations only to realize that RCC was the primary pathology, strikingly in the clavicle [2]. As seen in this patient, the initial impression given the patient's history was that of an orthopedic nature with RCC only being incidentally found on workup. This demonstrates the insidious onset of RCC and given its relatively common incidence when compared to other neoplasms, it should be kept in mind when young patients with no comorbidities present with sudden pathological fractures. 

## Conclusions

In conclusion, RCC is a common malignancy in which a significant number of patients have metastatic disease upon presentation and this can lead to initial confusion and delay in diagnosis. Hence it should be part of the differential diagnosis when investigating chronic bony pain and pathological fractures with concurrent nerve compromise, particularly in medically-free young patients.
